# Supplementary material for: Identification of photoperiod-induced specific miRNAs in the adrenal glands of Sunite sheep (Ovis aries)
Source: Front Vet Sci. 2022 Jul 22;9:888207. doi: 10.3389/fvets.2022.888207 (PMC9354845; doi:10.3389/fvets.2022.888207)
Supplement: Supplementary file 1 [file Data_Sheet_1.ZIP › Supplementary file/miRDeep2 file.zip!/miRDeep2 file/Novel_Structure/SPLP42A2/Novel_38.pdf]

Provisional ID	:	NC_019460.2_9322
Score total	:	2.4
Score for star read(s)	:	-1.3
Score for read counts	:	0
Score for mfe	:	2.1
Score for randfold	:	1.6
Score for cons. seed	:	
Total read count	:	741
Mature read count	:	733
Loop read count	:	0
Star read count	:	8

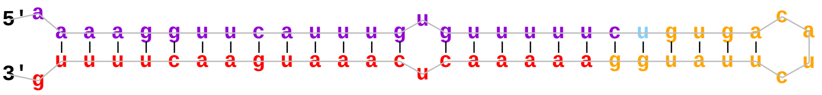

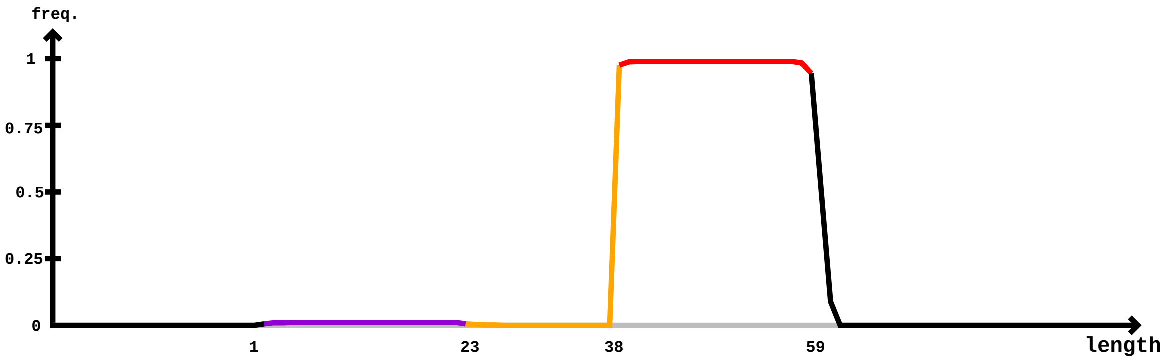

Star **Mature**

```
5'- gaaauaauacuggguuggccaaaagguucauuuguguuuuucugugacaucuuauggaaaaacucaaaugaacuuuugggccaacccaaugaaaagcuccaaucccuucac -3'   obs
    gaaauaauacuggguuggccaaaagguucauuuguguuuuucugugacaucuuauggaaaaacucaaaugaacuuuugggccaacccaaugaaaagcuccaaucccuucac       exp
    ..........((((((((((.(((((((((((((.((((((((((((....)))))))))))).))))))))))))).))))))))))).......................   reads   mm   sample
    ....................aaaagguucauuuguguuuuu......................................................................   2       0    seq
    ....................aaaagguucauuuguguuuuuc.....................................................................   2       0    seq
    .....................aaagguucauuuguguuuuu......................................................................   2       0    seq
    .....................aaagguucauuugAguuuuucu....................................................................   1       1    seq
    .......................agguucauuuguguuuuucugu..................................................................   1       0    seq
    .........................................................aaaaacucaaaugaacuuu...................................   4       0    seq
    .........................................................aaaaacucaaaugaacuuuu..................................   28      0    seq
    .........................................................aGaaacucaaaugaacuuuu..................................   1       1    seq
    .........................................................aaaaacucaaaugaacuuuug.................................   611     0    seq
    .........................................................aGaaacucaaaugaacuuuug.................................   1       1    seq
    .........................................................aaaaacucaaaugaacuuuuU.................................   1       1    seq
    .........................................................aaaaacucaaaugaacCuuug.................................   4       1    seq
    .........................................................aaaaacucaGaugaacuuuug.................................   2       1    seq
    .........................................................aaaaacucaaaugaacuuuuC.................................   1       1    seq
    .........................................................aaaaGcucaaaugaacuuuugg................................   1       1    seq
    .........................................................aaaaacucaGaugaacuuuugg................................   1       1    seq
    .........................................................aaaaacucaaaugaacuuuuCg................................   1       1    seq
    .........................................................aGaaacucaaaugaacuuuugg................................   3       1    seq
    .........................................................Gaaaacucaaaugaacuuuugg................................   1       1    seq
    .........................................................aaaaacucaaaugaacuuuuggU...............................   63      1    seq
    ..........................................................aaaacucaaaugaacuuuug.................................   3       0    seq
    ..........................................................aaaacucaaaugaacuuuuUg................................   4       1    seq
    ..........................................................aaaacucaaaugaacuuuuggU...............................   2       1    seq
    ...........................................................aaacucaaaugaacuuuuggU...............................   1       1    seq
```
